# The impact of technology use for care by informal female caregivers on their well-being: a scoping review

**DOI:** 10.1186/s13643-025-02817-z

**Published:** 2025-04-17

**Authors:** Andrés Aparicio, María Alejandra Inostroza-Correa, Paula Miranda, Carolina Salinas, Valeria Herskovic, Camila Chackiel, Catalina Alarcón

**Affiliations:** 1 Millennium Institute for Care Research (MICARE), Santiago, Chile; 2 Escuela de Trabajo Social, Pontificia Universidad Católica de Chile, Santiago, Chile; 3 Instituto de Sociología, Pontificia Universidad Católica de Chile, Santiago, Chile; 4 Facultad de Derecho, Pontificia Universidad Católica de Chile, Santiago, Chile; 5 Facultad de Ingeniería, Pontificia Universidad Católica de Chile, Santiago, Chile

**Keywords:** Gender, Care, Quality of life, Tools, Vulnerability

## Abstract

**Introduction:**

Informal caregiving roles are predominantly assumed by women, who often experience unique challenges related to physical, emotional, and social stress due to entrenched gender disparities. Within this context, the use of technologies to assist in caregiving tasks has become popular, and some evidence about their impact on caregiver well-being is available. However, significant research gaps persist. This scoping review was intended to assess the extent of literature examining the impact of technology use by informal female caregivers on their quality of life, to characterize existing research gaps, and to identify available evidence regarding gender-specific challenges.

**Methods:**

We searched for studies in English, Spanish, and Portuguese published in peer-reviewed journals since 2018. We included studies exploring how informal female caregivers use technology and how such use impacts their well-being. The studies included in the review analyze the impact of technology use on the physical, emotional, or material well-being of informal female caregivers. Sources were screened in stages by two independent reviewers; data were extracted from selected full texts, and results were integrated into a narrative summary.

**Results:**

A total of 14 studies were included in the review, highlighting a range of technologies such as health monitoring devices, communication platforms, and assistive aids. The review identified improvements in caregiver well-being related to reduced physical burden, enhanced emotional support, and increased social connectivity. However, significant research gaps were noted, particularly regarding the long-term effects of technology use, differences based on socio-economic contexts, and the limited inclusion of gender-specific analyses.

**Conclusions:**

This review supports the notion that technology use can positively impact the well-being of informal female caregivers, especially in terms of emotional and social support. Nevertheless, the review also found that in certain situations, technology can fail to improve or even worsen the quality of life of caregivers. However, the limited availability of studies with standardized quantitative measures, gender-specific data, and comprehensive assessments of long-term effects highlights areas for future research. Further exploration into diverse sociocultural contexts and empirical model development will be essential to better understand the nuanced ways in which technology use influences caregiver quality of life. These findings underscore the potential for targeted technology solutions to support informal caregivers, with implications for healthcare professionals and policymakers designing caregiver support initiatives.

## Background

The well-being of caregivers has become a prominent topic in recent studies on caregiving, especially for informal caregivers [4]. These informal caregivers are individuals who provide care without a contract or compensation and maintain a family relationship with the person they are caring for [13]. Recognizing that these caregiving roles are highly feminized activities [7, 36], primarily due to the persistence of traditional gender roles [2, 9, 12], research has shown that women engaged in caregiving are exposed to a multitude of risks. In this regard, significant deteriorations have been identified in indicators such as self-reported health [3, 12], physical health [6], and various dimensions of psychological and subjective well-being, as well as mental health [3, 4, 6, 32]. This impact may be particularly intense for female heads of [11, 23].

Within this context, the use of technologies to assist in caregiving tasks has become increasingly popular [1, 26]. Among these, the most common tools are electronic devices that facilitate communication and access to information such as speakers and voice recognition. There are also other categories of devices that allow remote monitoring of the individuals receiving care (telehealth) or support for vision and hearing (special screens, electronic narration, etc.), as well as aids for mobility [26]. In general, the use of these tools helps alleviate the physical and mental burden on caregivers and improves the satisfaction and fulfillment levels of their users while reducing the difficulty of specific tasks and activities [27, 29]. While technology offers promising benefits—such as enhanced communication and reduced physical burden—it may also introduce challenges including technical complexities, digital literacy barriers, and even increased caregiver stress when devices malfunction.

Nevertheless, significant research gaps persist, especially in terms of data production and availability. The literature tends to focus on countries with more resources, as the population with greater access to technology tends to have higher socio-economic status [26]. However, these socio-economic determinants are essential because informal caregiving is predominantly carried out by vulnerable women [13], where the mobilization of personal, family, and affective networks becomes more necessary to address the limitations of social protection [5]. This leaves a gap in our understanding of how these dynamics play out in the Global South as cultural, economic, and infrastructural differences may critically influence the accessibility and effectiveness of caregiving technologies. Given this context, the goal of this scoping review was to assess the extent of literature examining the impact of technology use by informal female caregivers on their quality of life to characterize existing research gaps and to identify what evidence is already available.

The general question that is addressed in this scoping review is *what is known about the impact of technology use by informal female caregivers on their well-being?* Additionally, the review pretends to answer the following sub-questions:

- Has any impact of technology use by informal caregivers on their well-being been identified? If so,
  - ◦ Can this impact be characterized in terms of the type of technology?
  - ◦ Can this impact be characterized in terms of sociocultural context?
  - ◦ Can this impact be characterized in terms of specific well-being domains?
- What models testing the relationship between technology use, well-being, and contextual variables have been reported?

## Methods

This scoping review was conducted following the JBI methodology for scoping reviews [30]. Associated with this review, there is a search protocol that was developed by the research team [20] prior to writing the article. This protocol can be accessed on the Open Science Framework platform, where it was registered: 10.6084/m9.figshare.24372490.v1.

### Eligibility criteria

#### Participants

This review was meant to include studies reporting results from samples of informal female caregivers of any age, or samples from caregivers in general if there are detailed results of a subsample of informal female caregivers. Studies focusing only on formal or male caregivers were excluded. However, we had to expand our inclusion criteria as, midway through the screening process, we realized most studies reviewed did not present separate results for men and women; in other words, gender was not considered as a criterion to evaluate the effects of technology use on well-being. As a result, we were forced to interpret this as a finding and to broaden the selection criterion to include studies where the sample predominantly consisted of informal female caregivers. While this adjustment allowed us to capture a broader spectrum of literature, it may have influenced the precision of gender-specific insights.

#### Concept

The studies included in the review must analyze the impact of technology use on the physical, emotional, or material well-being of informal female caregivers. We considered including studies that analyze the broader concept of quality of life if they report specific results on physical, emotional, or material well-being. The included studies must not only refer to technology of any kind, not restricted to digital tools, but also support tools, assistive devices, mobility aids, etc.

#### Context

We included studies from anywhere in the world, with a particular interest in studies reporting results from the Global South.

#### Types of sources

This scoping review encompassed various types of research designs, both experimental and non- experimental. It included randomized controlled trials, non-randomized controlled trials, before-and-after studies, and interrupted time-series studies. Moreover, we considered analytical observational studies, comprising prospective and retrospective cohort studies, case–control studies, and analytical cross-sectional studies, for potential inclusion. Descriptive observational study designs, such as case series, individual case reports, and descriptive cross-sectional studies, were also considered. In addition to quantitative research, qualitative studies focusing on qualitative data, including but not limited to phenomenology, grounded theory, ethnography, qualitative description, action research, and feminist research, could be included in our review. Systematic reviews were considered if they met the inclusion criteria and their question was relevant to the objective of this scoping review. We only considered studies published in peer-reviewed journals. Other kinds of documents were not considered due to resource constraints. We acknowledge that this approach might have excluded valuable gray literature, particularly from underrepresented regions, potentially introducing a bias toward studies from more established research contexts. This review included studies published in English, Spanish, and Portuguese to facilitate reading, screening, and extracting data. Studies published since 2018 could be included as we are interested in the current state of research and how it reflects contemporary models of well-being as part of the broader concept of quality of life. We searched WoS and Scopus databases.

### Sources of information

Two scientific bibliographic databases were used to compile sources of information: WoS and Scopus. These databases were chosen because they are universally known and widely recognized by the academic community. The most recent search was conducted on March 8, 2024.

### Search strategy

The search strategy focused on identifying studies published between January 1, 2018, and March 8, 2024. A preliminary search of WoS databases was conducted to identify studies related to the topic. From this result, we developed a full search strategy for WoS (see Appendix 1). We adapted the search strategy for each database. The reference list of all included documents was screened for additional studies. The languages included in the search were Spanish, English, and Portuguese. Additionally, the search was limited to studies indexed in the Science Citation Index Expanded, Social Sciences Citation Index, and Emerging Sources Citation Index. Only documents classified as “papers” in the document type were considered.

### Study/source of evidence selection

All identified citations were uploaded into Covidence [8], and duplicates were removed. Titles and abstracts were screened by two independent reviewers according to the inclusion criteria, and full texts for screened citations were then uploaded to Covidence. These full texts were assessed in detail by two independent reviewers to check whether they met the inclusion criteria. At any stage, disagreements between reviewers were resolved by discussion or by arbitration of a third reviewer. Reasons for excluding sources were recorded and reported in this scoping review. The results of the search and inclusion process are reported in a Preferred Reporting Items for Systematic Reviews and Meta-analyses extension for scoping reviews (PRISMA-ScR) flow diagram [35].

### Data extraction

Data extracted from sources were included in the scoping review by two independent reviewers using an extraction instrument designed by the reviewers. Extracted data included specific information about participants, concepts, contexts, methods, and key findings pertinent to the questions of the review. Disagreements between reviewers were resolved by discussion or by arbitration of a third reviewer. There was no need to contact the authors of papers to request data.

### Data charting process

Data extraction forms were created on Covidence. First, given the diversity of methods presented in the studies, we organized the main information from each article in Excel to develop criteria that would comprehensively cover all the information to be collected. The forms were therefore not adapted from the Covidence Data Extraction Form as expected but, instead, were constructed according to the characteristics of the selected studies (see Appendix 2: Data items). Once the forms were created, they were completed by two reviewers in Covidence to extract information and assess the quality of each article. During this process, it was not necessary to contact the studies’ authors to confirm data.

### Synthesis of results

We employed a thematic analysis to synthesize the selected studies, a flexible approach that allowed us to identify major themes and present them in a narrative format. Due to the heterogeneity of measurement types and statistical methods used across the articles, a quantitative meta-analysis was not feasible. Initially, four researchers independently reviewed and extracted data from each article using a structured extraction form. The team then met to compare findings, discuss key topics, and achieve investigator triangulation. Subsequently, all selected studies were uploaded into ATLAS.ti, where two researchers conducted iterative thematic coding based on the identified axes and two researchers reviewed the codes and themes. Throughout this process, findings were compared across studies and methodologies, thereby enhancing the robustness of the synthesized themes. Finally, the refined codes were grouped into emerging themes, providing a comprehensive narrative synthesis of the literature. The quality of the included studies was appraised using a custom instrument based on the JBI Critical Appraisal Tools [21]. The instrument has seventeen questions designed to assess the sample and interpretation bias, methodological quality, and ethical considerations with a score of 0–100; a study may be classified as high, medium, or low (see Apendix 3).

## Results

**Fig. 1:**
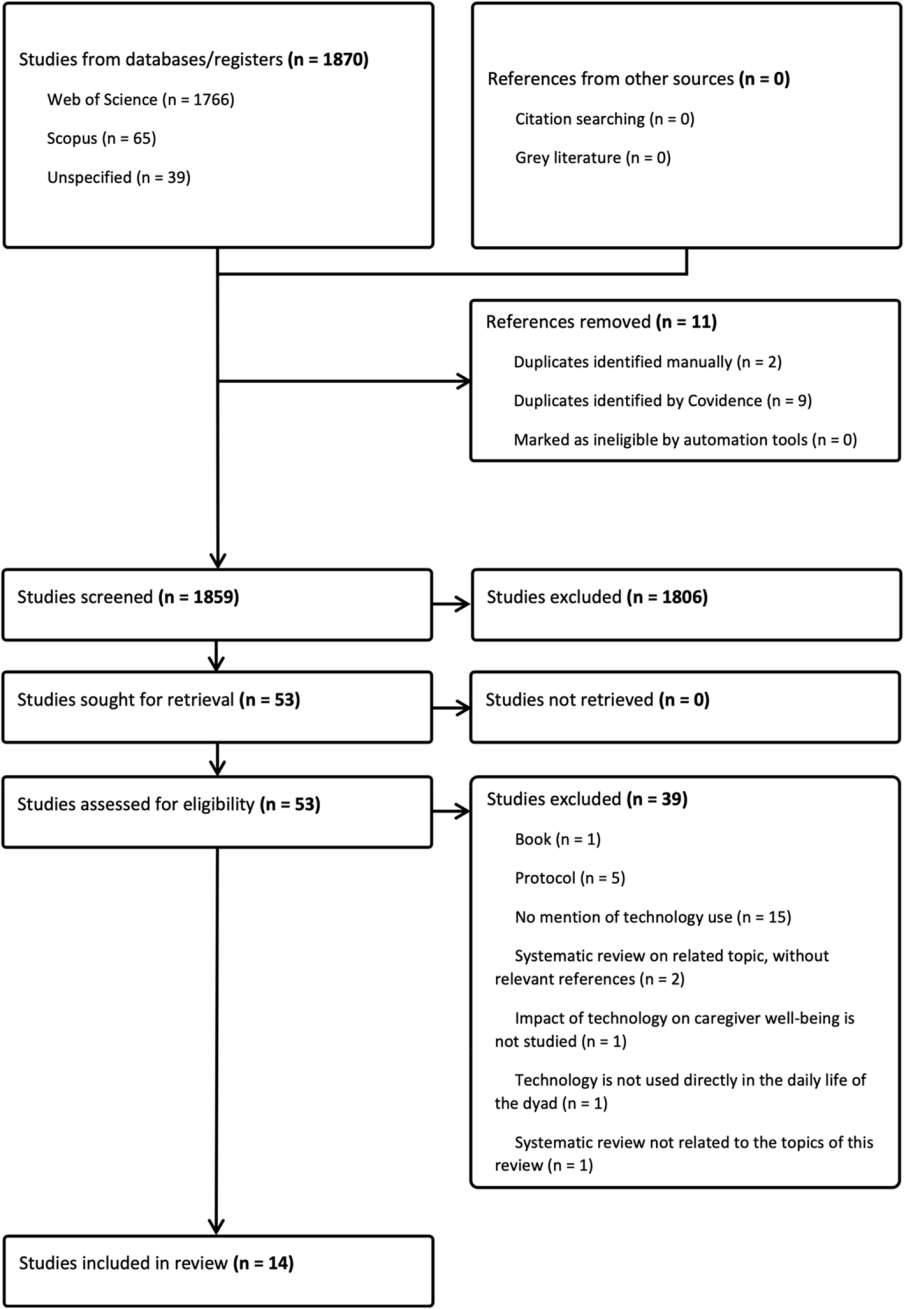
PRISMA ScR flowchart

The search process yielded 1870 studies, of which 11 were duplicates. After the screening process, we selected 14 studies for analysis (Fig. 1): three are qualitative [10, 15, 19], two used mixed methods [14, 25], and nine are quantitative [16–18, 22, 24, 28, 31, 33, 34]. Most of the studies originate from the Global North, with none from Latin America. A summary of their characteristics is presented in Table 1.

A critical appraisal of the quality of the included studies indicates several limitations. Only three studies are classified as high quality [25, 28, 33], whereas the rest are classified as medium quality. Only two studies [28, 33] have explicit measures to reduce sample bias. Regarding ethical considerations, 11 studies report being approved by an Ethical Review Board, whereas three studies either miss this information or the information provided is not clear enough [15–17]. Furthermore, only three studies discuss the ethical implications of their research [16, 25, 28].

Information was extracted and consolidated, and a thematic analysis was conducted. Four themes emerged from the thematic analysis: first, a characterization of caregiving support technologies; second, the different types of effects of technology use identified across the studies; third, an outline of potential mechanisms behind these effects; and finally, the challenges associated with technology use in the caregiving context. The next sections will present the synthesis of results for each theme.

**Table 1:** Selected studies characterics

	**Authors (year)**	**Country**	**Type and goal**	**Sample**
1	Griffiths, P. C., Kovaleva, M., Higgins, M., Langston, A. H., & Hepburn, K. (2018) [16]	USA	Quantitative. To examine the feasibility and efficacy of Tele-Savvy, an online version of the Savvy Caregiver Program, a psychoeducation program for caregivers caring for a person with dementia	*n* = 64 58 women
2	Elbalshy, M., Boucher, S., Crocket, H., Galland, B., MacKenzie, C., de Bock, M. I., Jefferies, C., Wiltshire, E., & Wheeler, B. J. (2020) [10]	New Zealand	Qualitative. To explore parental experiences of using a DIY Continuous Glucose Monitoring Solution (MM-CGM) in children and adolescents with Type 1 Diabetes	*n* = 12 11 women
3	Han, S. S., White, K., & Cisek, E. (2022) [18]	USA	Quantitative. To demonstrate that the use of assistive technology is feasible in the home and promotes effective behavioral interventions in family and domestic settings with dementia	*n* = 16 10 women
4	Holthe, T., Jentoft, R., Arntzen, C., & Thorsen, K. (2018) [19]	Norway	Qualitative. To examine the family caregiver roles and experiences with assistive technology as means of supporting people with young onset-dementia	*n* = 13 6 women (one couple cared for the same person)
5	Gallardo-Flores, A., Sánchez-Medina, J. A., & Fernández-Portero, C. (2018) [15]	Spain	Qualitative. (1) To determine whether adult and older informal caregivers’ perceptions of health, well-being, quality of life, and social support are influenced by their participation in a comprehensive technical training program composed of different disciplines. (2) To study the relationship between caregivers’ perceptions and their health, social relationships, quality of life, and social satisfaction	*n* = 13 13 women
6	Kato, N. P., Okada, I., Kagami, Y., Endo, M., Hatano, M., Ono, M., Jaarsma, T., & Kinugawa, K. (2018) [22]	Japan	Quantitative. (1) To evaluate the quality of life (QoL) of caregivers before and after LVAD implantation. (2) To identify factors associated with caregivers’ quality of life	*n* = 40 32 women
7	Pousada, T., Groba, B., Nieto-Riveiro, L., Pazos, A., Díez, E., & Pereira, J. (2018) [31]	Spain	Quantitative. (1) To determine the presence of burden among caregivers of people with neuromuscular diseases who use a wheelchair and whether this burden is associated with wheelchair use. (2) To establish whether caregiver burden is influenced by contextual factors (environmental and personal), including wheelchair use, in accordance with the International Classification of Functioning	*n* = 41 31 women
8	Guzmán-Parra, J., Barnestein-Fonseca, P., Guerrero-Pertiñez, G., Anderberg, P., Jimenez-Fernandez, L., Valero-Moreno, E., Goodman-Casanova, J. M., Cuesta-Vargas, A., Garolera, M., Quintana, M., García-Betances, R. I., Lemmens, E., Sanmartin Berglund, J., & Mayoral-Cleries, F. (2020) [17]	Spain and Sweden	Quantitative. (1) To analyze technophilia in people with dementia/mild cognitive impairment and their caregivers, and to determine the sociodemographic and clinical factors associated with technophilia. (2) To examine how this population uses smartphones, tablets, applications, and software to support memory, and to identify factors associated with the use of memory-supporting applications	*n* = 1086 741 women
9	Leszko M. (2020) [25]	Poland	Mixed. To investigate (1) what role does online communication play in the lives of female caregivers and (2) what impact does maintaining social contact through online communication have on caregivers’ well-being. (3) To identify barriers to Internet-based communication	*n* = 48 48 women
10	Flanagan, J., Post, K., Hill, R., & DiPalazzo, J. (2022) [14]	USA	Mixed. To determine the feasibility of a nurse-led walking intervention using wireless pedometers with informal caregivers of people with dementia	*n* = 32 27 women
11	Sriram, V., Jenkinson, C., & Peters, M. (2021) [34]	UK	Quantitative. (1) To investigate the types and use of assistive technology by caregivers in supporting people with dementia. (2) To examine the initial costs, ongoing monthly costs, and perceived value for money of assistive technology. (3) To explore the perceived impact of assistive technology on caregivers. (4) To assess the overall physical and mental health of caregivers of people with dementia who use assistive technology	*n* = 201 131 women
12	Sin, J., Henderson, C., Elkes, J., Cornelius, V., Woodham, L. A., Batchelor, R., Chen, T., Corredor, A. M., Coughlan, D., Dhital, R., Evans, S., Haider, B., Heathcote, J., Mansfield, S., O’Brien, A., Qassim, M., Sserunkuma, J., Travis, C. H., Williams, E., & Gillard, S. (2022) [33]	UK	Quantitative. To evaluate the impact of a multicomponent digital intervention called COPe-support on improving caregivers’ mental well-being and care-related outcomes	*n* = 407 330 women
13	Mahmood, A., Kim, H., Kedia, S., & Dillon, P. (2022) [28]	USA	Quantitative. To examine the association between the use of wearable devices and physical activity (PA) levels among informal caregivers in the United States	*n* = 1273 757 women
14	Kubo, A., Kurtovich, E., McGinnis, M., Aghaee, S., Altschuler, A., Quesenberry, C., Jr, Kolevska, T., Liu, R., Greyz-Yusupov, N., & Avins, A. (2024) [24]	USA	Quantitative. To assess the feasibility of conducting a cluster randomized controlled trial (RCT) comparing technology-delivered mindfulness-based intervention (MBI) programs against a waitlist control arm targeting advanced cancer patients and their informal caregivers	*n* = 39 31 women

### Characterization of care support technologies

#### Scope of technology use

The review of studies identified three main scopes of technology use in the caregiving context. Specifically, three types of users or recipients of technology use emerged: the dyad—comprising the caregiver and the care recipient; the caregiver—using technology on an individual level; and the care recipient—also at an individual level. Five studies reported technology use by the dyad [10, 17–19, 24], particularly for intervention purposes, although measurements were taken separately for each individual. In terms of individual technology use by the informal caregiver, seven studies focused on this context [14–16, 25, 28, 33, 34]. Finally, in two studies, the technology was used individually by the dependent person, regardless of whether the caregiver’s well-being was also measured,in one study, this technology was a wheelchair [31], whereas in the other, it was a left ventricular assist device (LVAD) [22].

#### Types of technology

Across the studies, a wide range of technologies was observed, differing in the type of support they provided and their intended use. Technologies were categorized into four types: health assistance and monitoring, communication and support networks, assistive technologies, and educational technologies. Each category is described below.

##### Health assistance and monitoring technologies

The technology in Kato et al. [22] was an LVAD implanted as a therapy for patients with heart failure, intended as either a bridge to a heart transplant or to help a weakened heart pump blood to the body. This longitudinal study included caregivers of patients using various LVADs, such as DuraHeart, EVAHEART, HeartMate II, Jarvik 2000, and HVAD.

Three other technologies focused on health monitoring. In Flanagan et al. [14], an intervention was conducted where informal caregivers went for walks with wireless pedometers, paired with an online Fitbit control panel to access data on daily steps, personalize activity information (such as total steps or calories burned), and join online communities. The intervention included receiving guided walking messages from a nurse, with options to choose the frequency and medium—email, text message, or phone call [14].

Elbalshy et al. [10] explored the experiences of parents of children and adolescents with diabetes using DIY continuous glucose monitors (CGM). This system, called MiaoMiao, is placed over the standard Flash Glucose Monitor sensor and uses near-field communication to read raw sensor data, transmitting it via Bluetooth to a paired smart device. It bypasses the official algorithm, processing data through an unofficial application [10].

In Mahmood et al. [28], the technology was focused on health monitoring, specifically exploring the effect of wearable technology for tracking physical activity and health on caregivers’ well-being. Physical well-being was examined in relation to electronic wearables like Fitbit, Apple Watch, or Garmin Vivofit, which track physical activity [28].

##### Communication and support network technologies

Three studies examined technologies aimed at enhancing communication and social support networks. Gallardo-Flores et al. [15] reported improvements in caregivers’ well-being following a program for informal caregivers, where one intervention component involved new communication and social network technologies. Caregivers used phones and social networks like WhatsApp to maintain their social support networks, relationships, and community participation, serving as tools to communicate with friends. The intervention led to an increase in caregivers’ frequency of contact with people in their networks [15].

The clinical trial by Sin et al. [33] included an intervention that fits into both communication and support networks and educational technology. This intervention involved COPe-support, an interactive multimedia platform on the Canvas web platform. The platform facilitated support networks among peers and professionals in a learning environment, whereas the control group only accessed passive informational resources, with no caregiver interaction [33]. Features included a peer forum to exchange views and support, a caregiver resource section with links to relevant external resources (such as legal and professional organizations, charities, books, and online information sites), and a web support link for participants to seek technical or emotional support as needed [33].

Leszko [25] studied the importance of online communication and maintaining social contact among wives who care for individuals with Alzheimer’s. Here, the technology involved social media sites, online support groups, Skype, WhatsApp, and similar internet-based tools for daily online communication with others, including friends, family, and support groups [25].

##### Assistive technology

Four studies focused on tools that fit into the emerging category of assistive technology, defined as any item, piece of equipment, product, or system used to increase, maintain, or improve functional abilities and independence in individuals with cognitive, physical, or communication challenges (The Audit Commission, 2004 in [34]).

In Sriram et al. [34], the study explored caregiver experiences using assistive technologies for people with dementia. A variety of assistive technologies were identified, including smartphones and tablets (the most used), video communication systems, dementia watches, stair lifts, electric toothbrushes, hoists, assistive robots, flood detectors, and robotic pets [34]. These technologies served functions related to managing daily expenses, personal care activities like eating, washing, dressing, and using the bathroom, outdoor mobility, financial management, indoor mobility, reducing caregiver burden, leisure, memory support, communication, and safety [34].

Holthe et al. [19] also focused on daily experiences with assistive technology among family caregivers of individuals with early-onset dementia. They considered assistive technology as supporting cognition and functionality and provided dyads with automatic calendars and kitchen timers [19]. Caregivers used a range of assistive technologies, including sensors, location devices, compensatory and easy-to-use devices, visual and verbal reminders, kitchen or coffee machine timers, automatic calendars, whiteboards, talking wristwatches, simple TV remotes, digital calendars (with remote control), manual message boxes, GPS, electronic door locks, color or text coding, alarmed medication dispensers, item locators, memory clocks, mobile phones, and a coffee maker-linked message box [19].

Pousada et al. [31] focused on caregivers of individuals with neuromuscular diseases who use wheelchairs daily and need assistance with daily life activities. The type of wheelchair used, whether manual or electric, was noted, and caregivers were asked about other assistive tools they used [31].

Lastly, Guzmán-Parra et al. [17] evaluated the dyadic use of specific applications on tablets, smartphones, or other touchscreen devices aimed at supporting memory. These technologies fit within the concept of information and communication technologies but specifically focus on aiding memory for people with dementia or cognitive impairment [17].

##### Educational technology

Four studies involved technologies related to learning, with tools or educational platforms for caregivers. Griffiths et al. [16] examined the feasibility and effectiveness of an online psychoeducational program, Tele-Savvy, for caregivers of individuals with dementia, aiming to teach how dementia affects behavior and functioning, as well as strategies to reduce caregiver burden and stress. The program required a tablet, computer, or mobile device,it included synchronous weekly group teleconferences and asynchronous videos presenting educational content. The program was complemented by weekly self-care classes—yoga, relaxation, and breathing exercises—along with a manual and workbook for practicing strategies to create a calm, high-quality environment for the person with dementia [16].

In Han et al. [18], educational technology was MapHabit, a visual mapping software application on encrypted tablets that used visual, audio, and text media to create step-by-step guides to help participants and caregivers structure and perform daily activities. Caregivers were trained to access a library of visual maps with options to customize them based on the care recipient’s needs [18]. Those who did not receive visual map training were instead trained to access a library of educational videos focused on Alzheimer’s, dementia care, and caregiver support. Both groups had discussion sessions with clinical coordinators [18].

In Kubo et al. [24], a mobile health (mHealth) intervention was conducted for advanced cancer patients and their informal caregivers, using the HeadspaceTM program, which offers guided meditation instructions through a website or mobile application. Caregivers participated daily, completing basic meditation courses, followed by a course specifically for those affected by cancer, teaching breathing exercises, journaling, and emotional visualization [24].

Lastly, the study by Sin et al. [33]—also mentioned under communication technologies—featured an interactive and multimedia digital psychoeducation intervention called COPe-support for family caregivers of individuals with psychosis. While the control group had access to passive online resources, the treatment group received this interactive learning environment on the Canvas web platform, which included psychoeducation sections on psychosis, treatment, and caregiving,information and exercises promoting well-being; and a forum for asking questions to medical experts and social service advisors [33].

### Types of technology effects

#### Contribution of technology to caregivers’ well-being

The reviewed literature suggests a common theme across multiple studies: technology use positively influences caregivers’ well-being and quality of life. Despite variations in the types of technology used and their contexts, a general outcome observed was an improvement in caregivers’ quality of life [16, 18, 19, 24, 34]. For example, Kato et al. [22] showed that quality of life scores significantly improved for caregivers of heart failure patients following the implantation of an LVAD. Similarly, although using a different type of technology, Leszko [25] found that higher levels of online communication (using communication technology) were negatively correlated with loneliness and depression and positively correlated with life satisfaction, all at statistically significant levels. Additionally, Elbalshy et al. [10] reported that all participants using a device to monitor glucose levels in children and adolescents with diabetes reported improved quality of life. Thus, regardless of the technology type, its use positively impacted caregivers’ well-being.

It is worth noting that technology use not only enhances quality of life but also has a bidirectional association: Guzmán-Parra et al. [17] demonstrated that higher levels of technophilia—the tendency to use more devices and technology—were associated with higher quality of life among informal caregivers. In this case, technology usage becomes the variable of interest, revealing that caregivers with a higher quality of life are more likely to use technology.

The question that arises concerns the mechanisms through which these improvements occur: what is it about technology that enhances well-being and quality-of-life scores? The following themes identified in the literature help clarify this phenomenon: improvements in physical health, sleep quality, reduction in caregiver burden, decrease in stress and/or anxiety, and increased prioritization of self-care.

##### Improvement in physical health

Flanagan et al. [14] conducted an intervention involving caregiver walking sessions aimed at increasing physical activity and, consequently, quality of life. The control group used pedometers, which significantly improved their well-being. According to participants, the pedometer technology positively influenced their motivation to walk [14]. The connectivity offered by the pedometer motivated participants to continue walking. Following the intervention, participants expressed that they joined the program to do something beneficial for themselves and noted that being part of this type of program helped them feel healthier [14].

Similarly, it appears that some health-monitoring technology motivates users to maintain an active and healthy lifestyle. Mahmood et al. [28] demonstrated this with wearable technology: “The results demonstrated a positive association between the use of wearables and levels of PA among informal caregivers in the USA. Therefore, efforts to incorporate wearable technology into the development of health-promoting programs or interventions for informal caregivers could potentially improve their health and well-being”.

##### Improvement in sleep quality

Elbalshy et al. [10] also found that the glucose monitoring device for children and adolescents with diabetes improved both the quantity and quality of sleep for both parents and children. The device reduced the frequency and need for nighttime blood glucose monitoring. One caregiver commented:

What it enables me to do is when I do wake up, I can just roll over, have a quick look, and go back to sleep. So you’re getting up less, possibly waking more, but that’s just a habit that you get into I guess and you’re not actually getting up out of bed [10].

The device, connected via Bluetooth to the parents’ phone, provided easy access to necessary information, which, while a small change, significantly facilitated caregiving tasks, including nighttime routines.

##### Reduction of caregiver burden

The assistance that technology provides in caregiving tasks is well-documented, both in caregiver burden scores and in reports from the caregivers themselves. First, Griffiths et al. [16] showed that following an online psychoeducation intervention for dementia caregivers, caregiver burden scores significantly decreased compared to pre-treatment levels. The authors attribute this change to the caregivers’ improved skills in handling their tasks [16]. Similarly, Han et al. [18] conducted a comparable psychoeducation program: the control group used educational videos for Alzheimer’s caregivers, whereas the treatment group used innovative visual mapping technology, which consisted of step-by-step images, audio, and videos on how to complete tasks in the caregiving context. The results showed that caregivers in the treatment group had reduced burden scores compared to those in the control group [18]. Burden scores were also analyzed in the study by Pousada et al. [31], which aimed to establish a relationship between caregiver burden and the use of the care recipient’s wheelchair, considering factors such as age and usage frequency. They found that the younger a person is when they begin using their first wheelchair, the greater the intensity of the caregiver’s burden,however, a higher frequency of use can positively impact burden levels.

The evidence regarding burden reduction is not limited to burden scores but includes direct reports from caregivers. In their study on general assistive technology use in dementia care, Holthe et al. [19] shared the experience of one caregiver who noted they had made some progress toward having an easier daily life, which they described as better than nothing.

##### Reduction of stress and/or anxiety

Some studies highlight how caregivers’ experiences with technology are characterized by reduced stress and/or anxiety. Among them are previously reviewed studies: both parents monitoring the glucose levels of their diabetic children [10] and caregivers using visual maps to learn about caring for Alzheimer’s patients reported lower levels of stress and anxiety [18]. For instance, all participants in the first study revealed a reduction in stress and anxiety. One parent mentioned that the monitoring device “has taken a lot of stress out of it, a lot of worry, a lot of thinking out of it because we can actually get real-time information especially if he is at school and we are at work. It has definitely helped us lead a more normal life” [10].

Another way in which technology reduces stress and anxiety is by helping caregivers manage the burdens and negative aspects associated with the caregiving role. For example, in the article by Leszko [25], a woman shared her experience of losing social contact with loved ones—an issue that will be addressed in the next section—due to the demanding nature of caring for her husband with Alzheimer’s. She explained how technology has helped her cope with this:

I wish I could have friends or family over for coffee or dinner but it’s difficult for me to focus on my husband and the guests at the same time. With Skype, I can talk with them in the evening when my husband goes to bed. It’s really convenient. My sister lives nearby but my brother lives in a different city. We can have a conference call and talk at the same time and I don’t have to take the train and drag him along. It’s less stressful. [25].

Lastly, and more evidently, stress and anxiety reduction is also seen in the intervention by Kubo et al. [24]. This intervention involved a mindfulness course for cancer patients and their informal caregivers, where technology played a key role: the treatment group completed the course via a smartphone app, whereas the control group attended the course through webinars. The results showed that after 6 weeks, there was a substantial reduction in anxiety. Additionally, participants reported that one benefit of the intervention was that it equipped them with tools to manage stress and anxiety in future situations [24].

##### Prioritization of self-care

Technologies related to monitoring, reporting, or controlling health status appear to encourage caregivers to pay attention to their own self-care. In the previously reviewed study on the walking program for caregivers, which involved using a pedometer, participants expressed increased awareness of their own health and self-care needs; the walks served as a motivating factor for this, even though caregiving responsibilities sometimes posed an obstacle to consistently engaging in the activity [14]. A similar effect was observed in the study by Mahmood et al. [28], which examined the use of wearable technology to track physical activity among informal caregivers. In this study, more than a quarter of the participants reported feeling completely or very confident in their ability to care for their own health, indicating that caregiving demands were not interfering with their ability to practice self-care, at least among those using such devices [28].

Finally, it is worth noting that self-care is not limited to physical activity, as highlighted in the previous studies. General aspects of self-care are also emphasized. In Gallardo-Flores et al. [15], perceptions of health, well-being, and quality of life were examined among female caregivers in Spain, including an intervention involving several sessions aimed at improving their well-being (some of which involved technology). After the program, the women caregivers reported learning the importance of self-care in a broad sense (one of the intervention’s objectives), realizing that caregiving tasks often push their own health into the background [15].

#### Strengthening support networks

Several studies highlight the role of technology in connecting caregivers, whether with friends and family or with support networks of other caregivers. This section seeks to emphasize the role of technology in strengthening support networks, as these technologies enable connections among users. Two main effects are recognized: improvement in social relationships and resources, and increased socio-emotional support through technology.

##### Improvement in social relationships and resources

One key aspect highlighted in the research is how technology promotes social connection and support among informal caregivers. In the study on the walking intervention for dementia caregivers, it was found that simply being monitored and supported by a nurse through a step counter created a sense of connection and access to social resources [14]. Participants valued the regular and empathetic communication provided by the nurses, which made them feel part of something larger and less isolated in their caregiving experience,some even noted that the desire for social connection was a motivator to join the study [14], which they felt they achieved after the intervention. Along the same lines, assistive technology also alleviates caregiver burden and strengthens relationships with care recipients [19]. In the context of early-onset dementia care, technological tools were found to simplify daily tasks and promote a safer environment for patients. This not only benefited care recipients but also strengthened the relationship between caregivers and those they cared for, fostering a sense of shared purpose and emotional relief [19].

Online communication has proven especially valuable for caregivers, providing a safe space to share emotions, obtain emotional support, and maintain social connections [25]. Through platforms like online support groups and messaging apps, caregivers can interact with others in similar situations, sharing experiences and providing mutual comfort [25]. This virtual connection not only alleviates feelings of loneliness but also strengthens caregivers’ social ties, allowing them to maintain meaningful relationships despite the time and space constraints imposed by caregiving responsibilities [25].

In conclusion, integrating technology into informal caregivers’ lives improves their quality of life and strengthens their social relationships and resources. From emotional connection through online platforms to the freedom and relief provided by monitoring apps, technology is understood not only as a practical tool but also as a means to foster human connection and mutual support in the context of informal caregiving [15], a topic that will be addressed in the next section.

##### Socio-emotional support through technology

The socio-emotional support that technology provides to informal caregivers has been a recurring theme in the reviewed studies. According to Flanagan et al. [14], various technological interventions and platforms not only offer practical assistance but also nurture the emotional needs of those caring for loved ones.

As mentioned previously by Flanagan et al. [14] regarding the walking intervention, participants emphasized how simply knowing someone was there to monitor and understand them fostered an invaluable sense of connection. This led to an improvement not only in their social resources but also in their emotional connection within the program. The study noted that caregivers valued sharing their progress through a pedometer and receiving flexible, empathetic communication from nurses, which motivated them to care for themselves and made them feel part of a supportive community [14]. Additionally, a pilot study on mindfulness-based interventions for advanced cancer patients and their caregivers demonstrated that technology offers not only practical tools but also a space to address caregivers’ emotional needs during challenging times, enabling relationships that provide emotional support [24].

There is also ample evidence regarding the capacity of online communication platforms to provide emotional support for caregivers. Leszko’s [25] study on online communication among caregivers of people with dementia shows that through online support groups, caregivers can share experiences, gain practical insights, and receive comfort from those who understand their unique challenges. This virtual connection not only provides valuable information for managing the illness but also validates caregivers’ emotions, allowing them to express concerns and receive support from people facing similar situations [25]. In contexts where time and responsibilities limit caregivers’ ability to seek in-person support, technology serves as an invaluable tool for meeting their emotional needs. Research on the perception of health, well-being, and quality of life among female caregivers [15] shows that platforms like WhatsApp allow caregivers to stay connected with loved ones and receive emotional support even when their schedules are too demanding for in-person meetings.

In summary, according to various researchers, technology plays a crucial role in providing the socio-emotional support needed in a demanding and potentially socially isolating caregiving context. Technological tools allow caregivers to feel connected, understood, and supported in their caregiving roles.

#### Negative effects and aspects of technology use

While positive findings from the reviewed studies highlight the benefits of technology for caregivers’ quality of life, well-being, and support networks, some studies also emphasize the “B side” of these aspects. In certain situations, technology can increase burden, stress, and anxiety, strain social relationships, and even worsen—or simply fail to improve—the quality of life over the intervention period. The following sections discuss each of these issues.

##### Increased burden

The burden experienced by informal caregivers from using assistive technology appears as a theme in some studies [19, 22, 31]. The information gathered reveals a complex interaction between technology and caregivers’ perceived burden, often differing based on individual circumstances and device characteristics.

For example, Holthe et al. [19] noted that delays in the availability of assistive technology can lead to missed opportunities for necessary support, increasing the risk that the device may no longer be effective when finally implemented, thus adding to the caregiving burden. Additionally, changes in daily routines, such as vacations or hospital visits, can disrupt the technology’s effectiveness, turning what was once helpful into a new burden for caregivers [19]. The complexity and learning curve associated with some assistive devices can also contribute to increased perceived burden [19], as caregivers may find it challenging to operate devices that were initially helpful, such as electronic calendars or remote controls for TVs, leading them to prefer more traditional, less stressful methods like paper calendars or manual controls, avoiding additional strain in their already demanding caregiving routine.

As mentioned in an earlier section, Pousada et al. [31] found that factors like the age at which a person begins using a wheelchair and usage frequency can influence the caregiver’s burden perception, highlighting the importance of considering individual characteristics in each caregiving situation [31]. In some cases, technology use may create obstacles rather than ease caregiving tasks.

##### Stress and anxiety

While assistive technology can offer potential benefits for informal caregivers, it can also become a new source of stress and anxiety. Griffiths et al. [16], in a study on an online program for dementia caregivers, reported that caregivers’ distress and discomfort increased after completing the program, although the increase was not statistically significant. This finding suggests that although technology provides valuable tools, its use may come with emotional challenges for caregivers. Holthe et al. [19] noted that prolonged delays in the installation, evaluation, diagnosis, and repair of assistive devices can be a significant source of stress and frustration for caregivers, affecting their perception of the technology’s usefulness [19]. Some caregivers also reported technical issues with assistive technology devices, which led to frustration and concern [19].

Leszko’s [25] study on online communication among dementia caregivers revealed that some participants experienced negative feelings, such as anxiety and worry when reading posts from other members. The potential for misunderstandings and conflicts within online groups also contributed negatively to caregivers’ emotional burden. Additionally, the pressure to actively participate in online communication could strain family relationships and generate further conflicts [25]. Similarly, Gallardo-Flores et al. [15] highlighted that caregivers often experience anxiety and stress due to concerns about not doing enough for the dependent person. These findings suggest that the emotional burden associated with caregiving may be exacerbated by the perception of failing to meet caregiving expectations.

##### Deterioration of social relationships

Findings also reveal concerns about the deterioration of caregivers’ social relationships associated with technology use. Flanagan et al. [14] noted that while caregivers had the option to connect online with others through a pedometer, many participants expressed reluctance due to a feeling of not belonging to a group that understood their situation [14], despite shared caregiving circumstances. Similarly, caregivers in Gallardo-Flores et al. [15] described how caring for a dependent relative impacted their ability to participate in social activities, leading to a reduction or disappearance of these relationships. Many recounted how activities they once enjoyed, such as meeting friends or participating in social groups, became limited or eliminated due to caregiving demands. This finding is consistent across multiple studies [14, 22, 31], but highlights that technology meant to facilitate communication and relationships can have negative effects on social connections and the emotional well-being of caregivers. One participant stated:

...I only talk by phone to my friends occasionally. I feel sad because we had a very close relationship, and now, I’m a little more distant... I used to clear my head with them... that gave me life [15].

These findings reveal how technology can have varied impacts on the same phenomenon: previously, technology was seen to strengthen social networks and relationships, yet it may simultaneously undermine them, affecting caregivers’ emotional well-being and quality of life by fostering a sense of social distancing.

##### Deterioration or lack of effect on quality of life over intervention time

Among the reviewed studies, caregivers reported experiences suggesting a tendency toward a decline or lack of significant effects on quality of life over the intervention period. The study by Gallardo-Flores et al. [15] noted that in female caregivers, quality of life tends to deteriorate over time, even with technologies intended to prevent this. The idea is that the intense commitment to caregiving for a dependent person often translates into giving up free time, leisure, and self-care, limiting caregivers’ opportunities to engage in social activities and maintain social relationships [15].

Several studies found no significant effects of technology on informal caregivers’ quality of life over time. For example, Flanagan et al. [14] observed no significant changes in well-being for the control and intervention groups in their study on a walking intervention for informal caregivers of people with dementia. Similarly, Kato et al. [22] and Sin et al. [33] did not find significant improvements in caregivers’ physical or mental quality of life over time, despite various interventions. Kato et al. [22], in a study on LVAD implantation for heart failure patients, found no significant changes in caregivers’ physical quality of life over the intervention period. Sin et al. [33] found that using COPe-support, an interactive online psychoeducation program for caregivers of people with psychosis, did not significantly improve caregivers’ mental well-being after 20 weeks. In summary, technology does not always guarantee sustained improvement in informal caregivers’ quality of life over the intervention period.

### Mechanisms

Another theme that emerged from the analysis of the fourteen studies was the identification of different mechanisms that enhance caregivers’ skills, facilitate their caregiving tasks, motivate technology use, and generate satisfaction with it, thus contributing to potential improvements in caregivers’ quality of life. This section presents ideas on how technology use can enhance caregivers’ quality of life by examining the characteristics of technologies used in the caregiving context.

#### Ways technology facilitates caregiving tasks

One way that technology use in the caregiving context facilitates caregivers’ tasks is through fostering autonomy. Pousada et al. [31] noted that the most used assistive technology to maintain autonomy for individuals with neuromuscular diseases is the wheelchair. However, the younger a person is when they start using a wheelchair, the greater the intensity of caregiver burden,on the other hand, more frequent wheelchair use can positively impact caregiver burden, possibly due to the autonomy this grants to the care recipient [31]. Autonomy also applies to caregivers themselves. For example, the glucose monitoring system used by families in Elbalshy et al.’s [10] study allowed them to make decisions regarding their child’s diabetes management, setting up alert systems without constant medical guidance. They were able to relax, as they could receive real-time information when their child was at school and they were at work, enabling a more normal life.

By incorporating assistive technology into daily routines, family caregivers sought to help people with dementia maintain habits through its use, encouraging them to act independently [19]. Autonomy was valued in various life areas, such as mobility, where “family carers valued that the person with YOD was able to go out alone and retain some freedom of movement” [19]. For instance, caregivers stated that a medication dispenser with reminders would support autonomy and safe self-administration for people with dementia, eliminating the need for nurses to visit to administer medication, which had caused stress, anger, and confusion due to unpredictable visits [19]. Indeed, Holthe et al. [19] provided some participants with medication dispensers with alarms for taking pills, and caregivers felt relieved of caregiving tasks once the person with dementia became familiar with the device, as they no longer needed to constantly call and check if medications had been taken. Similarly, watches that verbally announced the time when a button was pressed helped reduce questions, stress, and misunderstandings among people with dementia [19].

Thus, autonomy, particularly through memory support, is one way that technology facilitates caregiving tasks, as seen with medication dispensers with alarms [19]. Guzmán-Parra et al. [17] found that the use of memory-support applications on tablets or mobile phones by caregivers was not associated with gender but was more common among younger, more educated caregivers with higher caregiving burden. Additionally, Sriram et al. [34] analyzed data from 201 caregivers and found that smartphones and tablets were the most frequently used assistive technologies, mainly for memory support and reminders for caregivers.

#### Technologies that enhance caregiving skills

A second area of these mechanisms involves technologies that enhance caregiving skills, empowering caregivers in their role and enabling them to perform caregiving tasks more effectively. Often, this is associated with increased communication and information sharing among caregivers, facilitated by technology, or information directly provided by the technology itself.

Being informed and connected is crucial for informal caregivers. For example, Sriram et al. [34] found that, after safety, communication was the primary reason caregivers used assistive technology. Thus, increasing communication and information is fundamental for using technology. The desire for social connection often motivates participation in technology-based interventions. In the nurse-guided walking study, caregivers reported feeling connected and appreciated that someone cared about them, checked on them, listened to them, and just seeing the pedometer reminded them of this, making them feel good and motivated to walk [14]. Participants in this walking program felt motivated, believing it would make them healthier and better caregivers [14]. Similarly, older women caring for spouses with Alzheimer’s used online support groups to share emotions related to the caregiving burden, maintain a sense of social connection with other caregivers, and access information about the disease. They gained practical tips and effective solutions for specific behaviors through responses from other caregivers [25]. Parents monitoring their children’s glucose reported that real-time information on their mobile phones was more useful and informative than other methods, like finger pricking [10].

A lack of knowledge about the illness is a significant challenge caregivers face, alongside financial or emotional tensions, and many caregivers feel unprepared to provide optimal care [25]. Online groups, however, offered an opportunity to gain knowledge and exchange information with other caregivers, helping them feel better prepared for the progression of their husbands’ disease [25]. Participants in the intervention considered these online groups a valuable source of information,even though no one was there to guide them step-by-step, they had a way to support each other, learn from mistakes, and observe others’ experiences. This transition from feeling “in the dark” to understanding disease stages and expectations was valuable [25]. A specific benefit of online group communication was gaining knowledge about legal processes and seeking financial or professional health support, including required documents, wait times, and other details [25]. Online communication also helped caregivers stay connected with friends and family,for example, participants saw photos of their grandchildren or children and learned about important events in their lives through Messenger or Skype [25]. A motivating factor associated with communication is the ability of caregivers to release emotions. Participants in Leszko et al.’s [25] study stated that they often communicated online with other caregivers rather than family members because they wanted to vent their frustrations without judgment or unsolicited advice.

Focusing on becoming better caregivers, various technology interventions generated this feeling. Dementia caregivers who completed the online Tele-Savvy program showed significant improvements in self-reported caregiving competence [16]. Additionally, the results suggested that greater improvements in caregiver competence led to further reductions in caregiver burden [16], indicating that feeling like a better caregiver may be a mechanism by which technology use in caregiving improves informal caregivers’ well-being.

#### Satisfaction with technology use and motivations for its use

Appreciation for safety associated with technology use was evident in more than one article. For example, Sriram et al. [34] identified safety as the most frequent reason for caregivers’ use of assistive technology. Parents of children with diabetes reported peace of mind due to the security and convenience provided by the glucose monitoring device [10]. Additionally, both members of a dyad, in which one caregiver used a GPS to locate his wife in the city, were satisfied with how easy and effective the device was, highlighting that technologies supporting meaningful and safe activities, especially when the person with dementia was alone, were valuable to caregivers [19].

In addition to safety, there was a clear preference for easy-to-use technologies [19]. Caregivers valued assistive technologies that contributed to simpler and safer days for people with YOD [19]. In this regard, couples in Holthe et al.’s [19] study committed to a shared goal of preserving normal daily life, which improved relationship quality and provided relief, with small successes over time enhancing motivation. An example is parents of diabetic children who appreciated that the intervention system helped them learn about the disease and make independent decisions about blood glucose fluctuations, with some reporting that the device exceeded expectations, relieving stress and enabling a more normal life [10]. Most parents initially struggled with the setup but later appreciated features like a long-lasting, rechargeable battery and a reasonably sized device compared to others [10].

When incorporating assistive technology into daily routines, caregivers preferred devices that were user-friendly, as these were most successful, especially for people with dementia who could manage tasks like using a TV remote or checking the time with speaking clocks [19]. Participants also found GPS easy to use, relieving the worry of knowing the person’s location [19]. Some caregivers mentioned that certain assistive technology required many operational steps, making it more challenging to use successfully,for example, one husband who learned to use a digital calendar for his wife’s scheduling while he worked found it time-consuming and complex compared to a paper calendar, ultimately rejecting it due to its burden [19]. In general, issues arose when devices became too complex, as dementia progression often made their use too challenging [19]. A significant finding was that “the more sophisticated the technology, the more challenging it was to get it to function in everyday practice, and the more it depended on a committed caregiver” [19], p.6).

Similarly, in Kubo et al.’s [24] intervention, most participants reported that meditating daily with the program was not difficult, with time as the only barrier, and expressed high satisfaction with the program. This makes sense as common reasons for participation were an interest in mindfulness, anxiety management, stress reduction, and supporting a family member with cancer [24], showing that motivation paid off. Participants in the pedometer-guided walking intervention noted that nurse messages and calls were simple, time-efficient, and flexible and helped them feel connected [14]. Finally, the visual maps assistive technology intervention showed that caregivers who used them reported high satisfaction with the program and would recommend it, making it a viable form of assistive technology for home use and more accessible than other educational methods for dementia caregivers [18].

Ease of use and security provided by technology are common motivating factors in caregiving and help maintain caregiver commitment to adopting technology in daily routines. This is reinforced by other previously mentioned themes, such as the desire for information or communication and the sense of improving caregiving skills.

### Challenges of technology use

The use of technology in informal caregiving presents several significant challenges for caregivers, impacting both the effectiveness of technological tools and the emotional and practical well-being of caregivers. These challenges include age-related barriers, issues of reliability and technology management, feelings of misunderstanding, loneliness, grief, and the need to replace outdated devices. The following sections detail these challenges, organized by category.

#### Age as a barrier

Caregivers’ age can negatively influence their willingness and ability to use online communication technologies. According to Leszko [25], older caregivers tend to spend less time communicating online, limiting their access to virtual support networks and technological resources. This barrier can be especially significant in informal caregiving, where connecting with support networks offers emotional and practical relief. Limited interaction with these networks due to less familiarity with technology can increase isolation and burden among older caregivers. Additionally, resistance to using more advanced technologies may be higher in this group, reducing opportunities to benefit from digital interventions that could improve their well-being and the quality of care they provide.

#### Unreliable technology

The lack of reliability in some assistive technologies is a recurring issue in caregivers’ daily use. Holthe et al. [19] noted that some informal caregivers experienced unwanted sounds from assistive devices at inconvenient times, and their inability to resolve these issues led them to view the technology as unreliable. This perception not only generates frustration but may also reduce confidence in the continued use of these technologies. In contexts where technology is supposed to simplify daily life or reduce caregiving burden, such failures can have the opposite effect, increasing burden and stress rather than alleviating them. The resulting distrust may lead caregivers to avoid adopting new technologies, preferring traditional methods they consider safer, albeit less efficient.

#### Feeling misunderstood as a barrier

Many caregivers prefer to communicate online with other caregivers rather than family members to vent frustrations without judgment or unsolicited advice [25]. However, this preference also has its challenges. Online communication can lead to misunderstandings due to a lack of non-verbal cues and differing communication styles among participants. Leszko [25] documented that caregivers often avoid discussing their feelings with family to prevent judgment or advice they have not asked for, leading them to rely on online communities. However, these communities can be a double-edged sword: while providing empathy and support, they can also create misunderstandings or pressure caregivers to participate actively. These misunderstandings can create tension within online communities and exacerbate feelings of isolation and frustration, particularly when caregivers feel they cannot freely express concerns without being misunderstood.

#### The challenge of loneliness

Loneliness is a pervasive challenge for many caregivers, impacting various life areas, including motivation to adopt healthy behaviors. Flanagan et al. [14] documented that many participants reported feelings of loneliness and isolation, which made it challenging to stay motivated for physical activities, such as walking, even when supported by technologies like pedometers. While these devices are designed to encourage physical activity, they are not always effective at countering the profound sense of loneliness that some caregivers experience. A lack of connection with others, whether family, friends, or support communities, can inhibit not only physical activity but also the willingness to engage in other forms of self-care. This social disconnection intensifies the feeling of isolation and increases the emotional burden, which in turn negatively affects the quality of care provided.

#### The challenge of grief

Both active and anticipatory grief are significant challenges that caregivers face daily. Flanagan et al. [14] found that grief during caregiving directly impacted participants’ motivation to engage in physical activities like walking, although many also reported that once they did participate, they experienced emotional relief. However, this relief was not always enough to overcome the initial barriers that grief imposes. Supportive calls or text messages were identified as key elements in helping caregivers stay committed to these activities, serving as reminders and motivators to focus on positive aspects of their situation. Despite these interventions, grief remains a powerful barrier that can reduce the effectiveness of technologies designed to enhance caregivers’ well-being, as the emotional burden of grief often overshadows the benefits of technological tools.

#### There’s always a but…

Despite the potential benefits of technology, caregivers often face additional challenges that can reduce its effectiveness or even turn it into an added burden. Holthe et al. [19] highlighted that although assistive technologies are designed to facilitate caregiving, their successful implementation heavily depends on the caregiver’s commitment and skill. Many caregivers reported that using these technologies involved new tasks and habits that, instead of simplifying their lives, added complexity and frustration. The sophistication of some technologies can be counterproductive, as their operation requires a high level of caregiver commitment and competence, which is not always feasible given the demanding caregiving context. Additionally, the progression of conditions like dementia can render initially helpful technologies too complex or ineffective over time, leading caregivers to abandon them or seek less complex, though less efficient, alternatives.

#### Replacing the old

Replacing outdated devices with new ones also presents significant challenges. Sriram et al. [34] noted that certain assistive devices, such as pendant alarms and audiobooks, were frequently abandoned because people with dementia could no longer use them effectively. This abandonment reflects both the disease’s progression and the inherent difficulties in adapting to new technologies, especially when the care recipient’s cognitive abilities are declining. Elbalshy et al. [10] found that previous negative experiences with other CGM systems affected families’ willingness to switch to new devices, despite the potential improvements they offered. Resistance to change, along with difficulties in setting up and operating new technologies, can be a major barrier to adopting innovations that could enhance both the caregiver’s and care recipient’s quality of life.

#### Technological assistance

The incorporation of technology into caregiving often leads to substantial improvements in care quality, making the effort, time, and resources required to adopt new practices seem justified. The following quotes express this sentiment:

The FC [family caregivers] were particularly interested in AT [assistive technologies] that could sustain habits and support the person with YOD [young onset dementia] to cope better and act individually, and in devices that were easy to use [19]

Overall, participants described their experience of using MM-CGM [MiaoMiao CGM] as positive, expressing general satisfaction with the device. This was confirmed by the results of DTSQs [The Diabetes Treatment Satisfaction Questionnaires]. All participants noted positive aspects of remote monitoring and safety alarms with predictive trend arrows and graphs and associated increased awareness of hypo/ hyperglycemia [10]

However, in the case of neurological diseases, the degenerative nature of the conditions means that technology suitable for one stage may quickly become inadequate as the disease progresses. Sriram et al. [34] report that 81.2% of the sample abandoned the use of technologies such as pendant alarms or audiobooks because the person with dementia was no longer able to use them.

The way individuals receive treatment information is particularly important for caregivers of patients with rapidly deteriorating diseases, as interventions can seem inadequate or have negative effects before they are implemented. This is illustrated by the following quote from caregivers of people with dementia:

Well, this AT assistive technology] seems very nice, but it is too late for us to use this now. The dementia has progressed too far [19]

Complex information, diseases that create a sense of racing against time for caregivers, and high resource demands to access assistive technologies can lead to significant failures that damage the expectations caregivers had for the treatments they participated in. In these cases, support from a professional team is key to the success of the intervention:

The nurse called, she said don’t be so hard on yourself, set a smaller goal, walk 5 minutes every 2 hours or something like that, see if that works. That really helped . . . baby steps. That I could do.’ (Laura, 66 years, in [14]).

In summary, while assistive technologies hold substantial promise for supporting informal caregivers, the challenges highlighted here emphasize the need for thoughtful implementation and ongoing support. Addressing age-related barriers, enhancing the reliability and usability of devices, and providing comprehensive, accessible training are crucial steps in optimizing the benefits of technology for caregivers. Moreover, as caregivers often face unique emotional challenges, including feelings of isolation, grief, and the complexity of navigating evolving caregiving demands, it is essential that technology providers and healthcare professionals prioritize empathy, flexibility, and adaptability in their approach. Recognizing and mitigating these obstacles will be key to ensuring that assistive technologies contribute positively to the quality of life and well-being of caregivers and those they care for.

## Discussion

From the 14 studies reviewed, information was gathered to characterize support technologies in caregiving, the types of effects of this usage, the mechanisms of that impact, the relevance for key groups, and emerging themes. The answer to the main research question is affirmative: there is indeed an impact of technology use by informal caregivers on their well-being. Furthermore, it was possible to characterize the types of technology used in the caregiving context, addressing the first research sub-question. The types of technology were classified into four categories. The first category, health assistance and monitoring, includes ventricular assist devices, wireless pedometers, CGM, and wearable electronic devices for tracking physical activity [10, 14, 22, 28]. The second category includes technologies for communication and support networks: phones, social media (e.g., WhatsApp), and interactive, multimedia platforms for peer and professional support [15, 25, 33]. The third category encompasses general assistive technology: smartphones, tablets, video communication systems, tracking devices, wheelchairs, etc. [17, 19, 31, 34]. The fourth category is educational technology, including online psychoeducational programs, visual mapping applications, mindfulness apps, and interactive digital psychoeducation platforms [16, 18, 24, 33]. Besides this categorization, three areas of technology use were identified. One area was the dyad (caregiver and care recipient): technologies used jointly by the caregiver and the recipient [10, 17–19, 24]. Another area involved individual caregivers: technologies used exclusively by caregivers [14–16, 25, 28, 33, 34]. The third area was for care recipients only: technologies used exclusively by those receiving care [22, 31].

In relation to the sub-question on the impact of sociocultural context, there was no information to address it. This may be because, as shown in Table 1, nearly all studies came from Western countries or the Global North, with little attention given to the Latin American context, which was of initial interest. Future studies could delve further into the context of technology use, given the possible importance of sociocultural context and type of technology on observed impacts.

Regarding the characterization of this impact across different well-being domains, emotional well-being was predominant, with physical well-being considered in some studies, and almost no explicit mentions of material well-being. The types of effects of technology use for caregivers can be summarized across various dimensions or indicators of contributions to quality of life and well-being. Studies show that different technologies improve informal caregivers’ quality of life [16, 18, 19, 24, 34]. Furthermore, communication and support network technologies significantly reduce loneliness and depression [25]. Reduced anxiety and stress were common when incorporating certain technologies,for example, technologies that provide educational and emotional support help reduce caregiver burden and stress [16, 24]. Additionally, health-monitoring technologies motivate a healthy lifestyle, specifically pedometers and wearable electronic devices that encourage caregivers to maintain physical activity, improving their overall health and physical well-being [14, 28]. Along similar lines, glucose monitors and other continuous monitoring devices help improve sleep quality for both caregivers and care recipients [10]. Finally, one-way well-being was enhanced was through fostering self-care prioritization among caregivers and helping them feel more skilled, for instance, through educational and mindfulness programs that promote self-care among caregivers [18, 24].

Nevertheless, while many studies report benefits, studies also present mixed or negative outcomes. Several studies highlighted cases where technology increased the burden on caregivers due to technical difficulties or required extensive training [19, 22, 31]. Technology use can also become a new source of stress for caregivers [16, 19, 25]. This critical comparison suggests that the efficacy of technology in caregiving is contingent on both its design and the broader socio-economic and cultural context.

As for the research question about identifying models that demonstrate the relationship between technology use, well-being, and contextual variables, this question could not be fully answered due to the nature of the reviewed studies. Additionally, one study explored the inverse relationship, using technology use as the dependent variable rather than the independent variable. Moreover, the methodological techniques employed varied considerably, with some studies using mixed or qualitative approaches, rather than only quantitative—within quantitative studies, analysis methods also varied, likely due to the exploratory nature of the topic. Some findings indicated that while several models included gender as a control variable, distinguishing caregivers as men or women, results were not generally analyzed separately or used for interaction effects, making it difficult to address the research problem for the specific population of female informal caregivers for dependents. In this regard, future research could focus on a review specifically of models, and develop models based on empirical data to study this phenomenon, further exploring whether there is directionality in the relationship between technology use and informal caregivers’ well-being.

Finally, these results are relevant to three key groups, in addition to decision-makers who can influence caregiver support programs and policies. Health monitoring, educational, and communication technologies are especially relevant to the target population of informal caregivers, providing physical, emotional, and educational support. Second, these findings are significant for care recipients, those in dependent situations, as assistive technologies such as wheelchairs and ventricular assist devices are crucial for their mobility and health. The use of technology can improve relationships with caregivers and foster a better caregiving environment as caregivers acquire skills. Third, health professionals find relevance in these results, as technological interventions can be integrated into support programs to improve the quality of life for both caregivers and care recipients. Lastly, some emerging themes from the research reveal a wide range of technologies, from advanced medical devices to mobile applications and online platforms. Additionally, there is a diversity of effects of technology use on informal caregivers—mostly women—including physical, emotional, social, and educational benefits.

## Limitations

This scoping review offers valuable insights into how technology impacts the well-being of informal female caregivers, but several limitations should be acknowledged. First, a primary challenge encountered was the lack of explicit gender-based data in most studies. Although this review focused on female caregivers, many studies did not differentiate their findings by gender, which limited our ability to fully understand the unique impact of technology on female caregivers specifically. As a response, we expanded our inclusion criteria to encompass studies where female caregivers were predominant; however, this adjustment may have introduced a degree of bias. Future research would benefit from collecting and analyzing data with a gender-specific approach to offer a clearer understanding of the distinct needs and experiences of female caregivers.

Another limitation was the methodological diversity across the included studies, which made direct comparisons difficult. The review incorporated studies employing qualitative, quantitative, and mixed methods approaches, reflecting the complex nature of the topic. However, this diversity also presented a challenge, as there were no consistent measurement tools or standardized outcome variables, such as specific well-being scales, to allow for meaningful quantitative synthesis. This variability limited the potential for drawing generalizable conclusions. Standardized metrics and more rigorous methodological designs in future research could improve comparability and the robustness of findings in this field.

A further limitation was the geographical scope of the studies included. The majority of research originated in the Global North, with limited representation from the Global South, especially Latin America. This geographical imbalance may restrict the applicability of the findings to other socio-cultural contexts where caregiving norms, technology access, and support systems differ significantly. To address this gap, future studies should prioritize the exploration of technology’s impact in diverse cultural and socio-economic settings, as such factors may influence caregivers’ experiences and the adoption of technological interventions.

Additionally, the review highlighted the limited focus on the long-term effects of technology use among caregivers. Many studies included short-term interventions, and while they showed positive immediate effects on caregiver well-being, the sustainability of these benefits over time remains uncertain. The lack of longitudinal data makes it challenging to understand the enduring impact of technology in caregiving, especially in situations involving chronic stress or prolonged caregiving responsibilities. Longitudinal studies are therefore essential to assess the long-term implications of technology on caregiver well-being.

Lastly, while the review reveals the positive impacts of technology on caregivers, the studies did not adequately address the technological barriers caregivers might encounter. Key issues such as device reliability, ease of use, accessibility, and affordability were not thoroughly explored in most studies. Understanding these practical barriers is crucial to ensure that technological interventions are both beneficial and accessible to caregivers across different settings and income levels. Future research should delve deeper into these barriers to provide more comprehensive and actionable insights into the integration of technology into caregivers’ routines.

## Conclusions

This scoping review highlights that the use of technology can have a significantly positive impact on the well-being of informal female caregivers by supporting various aspects of their physical, emotional, and social health. Across the studies reviewed, it was found that technologies such as health-monitoring devices, communication platforms, assistive aids, and educational tools help improve caregivers’ quality of life by reducing stress, enhancing autonomy, and fostering emotional support.

A primary finding of this review is the strong impact of technology on caregivers’ emotional well-being. Communication technologies, including social media, messaging platforms, and online support groups, help reduce loneliness and feelings of isolation. These tools create opportunities for caregivers to connect with peers, share experiences, and receive essential emotional support, mitigating the social isolation that often accompanies caregiving roles. Through these networks, caregivers also gain access to valuable resources and practical advice, which further contributes to their sense of well-being.

Technology also positively influences caregivers’ physical health. Wearable devices, such as pedometers and fitness trackers, encourage physical activity, helping caregivers maintain a healthy lifestyle, whereas health-monitoring devices reduce the physical demands of caregiving by allowing caregivers to remotely monitor and manage aspects of their loved ones’ health. Moreover, continuous monitoring devices, such as glucose monitors, help improve sleep quality by alleviating the need for constant supervision. These health-focused technologies thus play a key role in reducing caregivers’ physical strain and promoting self-care.

In addition to providing emotional and physical support, technology fosters autonomy and self-care. Assistive devices, such as medication reminders, mobility aids, and memory-support tools, empower caregivers to manage tasks more efficiently while enabling care recipients to perform certain activities independently. This enhances the caregiving environment by reducing caregiver burden, promoting the independence of those receiving care, and fostering a healthier dynamic between caregivers and their dependents.

Technology may also affect negatively the well-being of caregivers. Care should be exercised when designing tools for caregiving to avoid increasing the burden of caregivers, provide technical support to decrease stress and anxiety due to the use of technology, and include contextual variables in the design of new technologies.

The review underscores a need for more inclusive research that considers diverse socio-cultural contexts. Most studies included in this review were conducted in Western, high-income countries, which may not fully capture the varied experiences of caregivers from different backgrounds and socio-economic statuses. The limited representation of studies from Latin America and the Global South highlights a gap in understanding how cultural, economic, and social factors influence the effectiveness of technology in caregiving. Additionally, few studies provided gender-specific insights, making it difficult to fully assess the unique experiences of female caregivers. Addressing these gaps in future research will offer a more comprehensive understanding of how technology can best support informal caregivers in various cultural contexts.

The findings of this review have practical implications for policymakers, healthcare professionals, and technology developers. There is a clear need to make technologies that are accessible, affordable, and user-friendly for informal caregivers, particularly those in economically vulnerable situations. Policymakers should consider incorporating technology solutions into caregiver support programs, ensuring these tools are designed to meet caregivers’ unique needs. Healthcare professionals also play a critical role in guiding caregivers towards appropriate technologies, helping them adopt tools that can improve their well-being and caregiving capabilities.

Future research should aim to address the limitations identified in this review, focusing on the long-term effects of technology use, socio-cultural factors, and gender-specific impacts. Moreover, the development of standardized, quantitative models that explore the relationship between technology use and caregiver well-being would advance the field, offering more consistent metrics to assess outcomes. Addressing practical barriers, such as device affordability, accessibility, and reliability, will also be crucial to ensuring that technology provides meaningful, sustainable support for caregivers in diverse settings.

In summary, while technology holds significant promise for improving the well-being of informal female caregivers, it is essential that future research and policy efforts consider the complexities of caregiving contexts. Technology that is thoughtfully designed and supported by structured interventions can make a profound difference, enhancing caregivers’ quality of life and promoting sustainable caregiving practices.

## Data Availability

All data and materials are available upon request.

## Appendix 1: Search query for WoS

(technolog* OR support* device* OR support* tool* OR support* system* OR assistive OR assistive device* OR assistive tool* OR assistive system*)

AND

(quality of life OR quality-of-life OR wellbeing OR well-being)

AND

(care*)

AND

(gender OR wom?n OR female)

## Appendix 2: Data items

**Table 2:** Data items

Variable	Indicators/attributes of the indicator
*General information*	
Reference	
Title	
Corresponding author	
Country where the study was conducted	
Notes	
*Characteristics*	
Method	
Research objective	
Data origin	Sample data collection Secondary data source Other
Research type	Quantitative Qualitative Mixed Other
Study type (quantitative component; mark all that apply)	Randomized controlled trial Non-randomized controlled trial Longitudinal study Pre-post without control Descriptive Correlational Not applicable (qualitative research only) Other
Study type (qualitative component; mark all that apply)	Focus groups Interviews Not applicable (quantitative research only) Other
Description of analysis methods	
Year of research	Year the research was conducted, not the publication date of the article
IRB approval	
Research funding	
Potential conflicts of interest	
*Participants*	
Sample description	
Inclusion criteria	
Exclusion criteria	
Recruitment method	In-person Phone Email Social media Health centers Foundations Other
Sample characteristics (for three subgroups informal caregivers, other groups, general)	Size Average age Weekly caregiving hours (average) Family income N/A
Relationship between informal caregiver and care recipient	Spouse Partner Daughter Mother Grandmother Other family Friend Neighbor Other relationship
*Technology*	
Technology type	Digital Analog
Technology description	
Technology use description	
Technology use	Frequency of use (in reported unit) Average usage hours
*Quantitative results*	
Reports separate results for men and women?	Yes No N/A
Outcome variables (PRE or single measurement) (up to 10 variables)	Variable name Average Standard deviation
Outcome variables (POST or N/A) (up to 10 variables)	Variable name Average Standard deviation
*Qualitative findings*	
Reports separate results for men and women?	Yes No N/A
Description of findings	
*Data extraction refinement*	
Relevant observations for the process	

## Appendix 3: Quality appraisal questions and scoring procedure

The study quality appraisal instrument has seventeen questions:

1. Is the research objective clearly described?
2. Are the method(s) used in the research appropriate to achieve the research objective?
3. Is the sample adequate for answering the research objective?
4. Was the sample selected to avoid biases in the results or findings?
5. Does the method used to recruit the sample ensure that biases which could have affected the results or findings are avoided?
6. Is there a discussion of potential biases due to the selected sample?
7. In the case of research with a control group, was the assignment of subjects to groups random?
8. In the case of research with a control group and non-random assignment, is the method of selection free from biases that could influence the results or findings?
9. In the case of the quantitative component, do the selected variables (both independent and dependent) relate to the research objective?
10. In the case of the quantitative component, are the analyses used adequate to meet the objective?
11. Was the research approved by an Ethics Committee?
12. Is there a discussion of the ethical implications of the research?
13. Is there a discussion of the ethical implications of the research?
14. In the case of the quantitative component, are the results of the analyses properly presented?
15. In the case of the qualitative component, do the findings align with the research objective?
16. In the case of the qualitative component, do the findings stem from the methodology used for analysis?
17. Is there a discussion of the results or findings? for 14 s

These questions are scored with 1 if the answer is Yes and 0 if the answer is not clear or there is no information. These scores are added, and the total is divided by the potential maximum score depending on the type of study and the presence of a control group (9 general questions, 4 questions regarding a quantitative component, 2 questions regarding a qualitative component, and 2 questions about the control group if present). Studies are then classified according to these 0–100 scores as high (≥ 80), medium (< 80, > 50), or low quality (< 50).

## Abbreviations

CGM: Continuous glucose monitors
LVAD: Left ventricular assist device
mHealth: Mobile health
PRISMA-ScR: Preferred Reporting Items for Systematic Reviews and Meta-analyses extension for scoping reviews
WoS: Web of Science
